# Empirical Study of Data Sharing by Authors Publishing in PLoS Journals

**DOI:** 10.1371/journal.pone.0007078

**Published:** 2009-09-18

**Authors:** Caroline J. Savage, Andrew J. Vickers

**Affiliations:** Department of Epidemiology and Biostatistics, Memorial Sloan-Kettering Cancer Center, New York, New York, United States of America; The Cochrane Collaboration, Germany

## Abstract

**Background:**

Many journals now require authors share their data with other investigators, either by depositing the data in a public repository or making it freely available upon request. These policies are explicit, but remain largely untested. We sought to determine how well authors comply with such policies by requesting data from authors who had published in one of two journals with clear data sharing policies.

**Methods and Findings:**

We requested data from ten investigators who had published in either PLoS Medicine or PLoS Clinical Trials. All responses were carefully documented. In the event that we were refused data, we reminded authors of the journal's data sharing guidelines. If we did not receive a response to our initial request, a second request was made. Following the ten requests for raw data, three investigators did not respond, four authors responded and refused to share their data, two email addresses were no longer valid, and one author requested further details. A reminder of PLoS's explicit requirement that authors share data did not change the reply from the four authors who initially refused. Only one author sent an original data set.

**Conclusions:**

We received only one of ten raw data sets requested. This suggests that journal policies requiring data sharing do not lead to authors making their data sets available to independent investigators.

## Introduction

Technology has dramatically improved the ways in which data can be stored, analyzed and disseminated. The Internet facilitates almost instantaneous access to data and information, and now plays a key role in many fields of medical research. Researchers in genomics, for example, rely heavily on publicly available resources such as GenBank, a repository of annotated DNA sequences. Many journals now state that submission of original data, such as microarray results, into appropriate repositories is a requirement for publication[1].

Following the notable strides of genomics, and other fields such as the open source movement in software, there has been a surge of awareness regarding data sharing in the biomedical field. The NIH recently declared that the sharing of data is essential for translating research into knowledge and products that improve health[2]. As such, all investigators seeking more than $500,000 in grant support per year are now required to include a plan for data sharing[3].

Several journals now require authors to share their raw data sets as a condition of publication. The Public Library of Science (PLoS) Journals, a collection of open access journals, specifically states that open access applies to both the scientific literature and the supporting data: “publication is conditional upon the agreement of authors to make freely available any materials and information associated with their publication that are reasonably requested by others for the purpose of academic, non-commercial research.”[4] Several other highly regarded journals, such as Nature and Science, have also established clear data sharing requirements[5], [6].

Unfortunately, the specific mechanisms for data sharing are often unspecified and the implementation of such policies largely untested. Very few journals have an explicit statement regarding how their data sharing policies are enforced, and thus it is unclear what options are available to investigators who encounter authors who are unwilling to share. To study how well authors comply with data sharing requests, we attempted to acquire original data sets from several researchers who had published in journals with explicit data sharing policies.

## Methods

We chose to make our requests from authors who had published in PLoS journals due to their exceedingly clear data sharing policies: all data relevant to publication should be deposited in an appropriate public repository, if appropriate, and if not, “data should be provided as supporting information with the published paper. If this is not practical, data should be made freely available upon reasonable request.”[4]

Ten papers from two PLoS publications, PLoS Medicine (n = 4) and PLoS Clinical Trials (n = 6). None included raw data as part of the supporting information. For all papers, the data requested would have allowed us to test a specific pre-specified hypothesis about prediction modeling, a scientific interest of the senior author. Our requests encompassed a wide range of diseases (cancer, malaria, HIV and others) and study designs (randomized controlled trials, case-control and cohort studies). We do not think it is plausible that our selection of papers amenable to prediction modeling could represent a selection of authors importantly more or less likely to share data than a random selection from PLoS publications. Data requests were emailed by CS to the corresponding author listed on the manuscript. Requests were similar in all cases: the stated reason was out of personal interest in the topic and the need for original data for master's level coursework.

With respect to ethical considerations, we pre-specified that any request must not create undue work for the investigators and that any data received as part of a successful request would be analyzed as planned, the results of which would be sent to the original investigators. In the event that we did obtain data, we would not guarantee to publish, but would promise a co-authorship on anything that was published.

If the response to our initial request was “no”, the stated reason was documented. A second request was then made by AV, who identified himself as an Editorial Board member for PLoS ONE. The email included an explicit reminder of PLoS's editorial policies, and stated clearly that, as the authors had published in a PLoS journal, they were required to provide free and open access to data sets used for analysis. The response to the second request was also documented. If we did not receive a response to our initial request, a second request was sent after one month.

## Results

**Figure 1:**
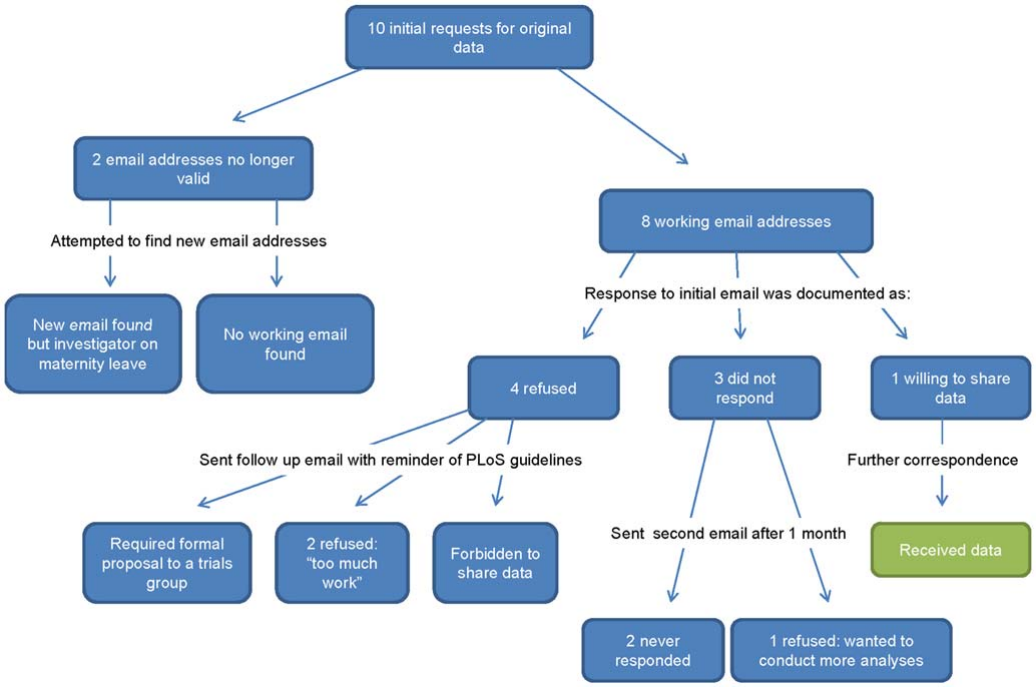
Summary of responses to the 10 initial requests for raw data.

We emailed initial requests for original data to ten corresponding authors. The responses are summarized in Figure 1. Two corresponding authors' email addresses listed on the paper were no longer valid as they had changed institutions since publication. After extensive investigation, we were able to track down an updated email address for one author; however, she was away on maternity leave and we have been unable to establish contact. An updated email address for the second author was never found. Of the remaining eight investigators with functioning email addresses, four authors replied that sharing their data was not possible, three authors did not respond, and another asked for further details regarding our request.

Of the four authors refusing to share their data in response to our initial request, two authors did not give a reason, one stated he was currently too busy, and the fourth said that he no longer had jurisdiction over the data as he had changed institutions. We sent a follow up email to these four investigators and reminded them of PLoS's data sharing policies. From the two authors who initially refused without providing a reason, one author said that we could submit a formal and lengthy proposal to the appropriate trialists' group; the other apologized that he had not been aware of the journal's data sharing policy and, as he was forbidden to pass the data on to third parties, he would not have published in PLoS had he been aware of this requirement. To the investigator who said he was currently too busy, we responded that we could wait to receive the data; his response was that it was simply too much work. Our request to the investigator who had changed institutions was forwarded to the appropriate researchers still working at the institution. They claimed it would take too long to organize and annotate the data set and would be too much work.

Three authors simply did not respond to our initial request. After a follow up email to these authors, one author replied that he was in favor of sharing data in general, but wished to conduct more analyses before sharing. We did not receive a reply from the other two authors.

One author replied to our initial request by asking for more information about our proposed analysis. After correspondence to discuss our analytic plan and the particular variables needed, we received a well annotated dataset within a few hours.

## Discussion

We requested raw data from ten corresponding authors and received only one data set. Although our sample was small, our results are clear: explicit data sharing policies in journals do not lead authors to share data. Our initial intention was to see if the rates of data sharing were any higher in journals with data sharing policies compared to those without. However, our initial results were sufficiently clear that a comparison was deemed unnecessary.

We are aware of only one prior study that described the difficultly of obtaining original data sets from published authors. Wicherts et al. wrote to more than one hundred authors who had published in American Psychological Association (APA) journals to request data for reanalysis[7]. They experienced similar difficulty in obtaining original data, receiving only one quarter of the requested data sets. Although our study was considerably smaller, it is importantly different for three reasons. First, the APA's policies are not as explicit as those from PLoS. The APA's guidelines state that authors should share their data “provided that the confidentiality of the participants can be protected and unless legal rights concerning proprietary data preclude their release,” a stipulation that may have permitted many authors to avoid sharing under the guise of patient confidentiality or legal rights[8]. Second, APA policies don't provide an incentive for original authors to share data, as data can only be used “to verify the substantive claims through reanalysis.” [8] Thus original authors stand only to lose by having their conclusions challenged. In contrast, we asked investigators for data not to challenge their original conclusions, but to test a new hypothesis. Third, the majority of data sets requested by Wicherts et al. were survey research; we requested data from medical studies and clinical trials - research that has a direct and immediate relevance to individuals suffering ill-health.

We acknowledge that there are numerous real and perceived impediments to sharing raw data. One of us has previously written extensively on this issue[9]. Concerns about patient privacy are frequently cited, although data from most studies can easily be coded in such a way as to ensure subject's anonymity. There are also concerns about authorship and future publishing opportunities, and a natural desire to retain exclusive access to data that may have taken many years of hard work to collect. We have published some simple guidelines to protect the rights of investigators to exploit their data, such as an embargo period between publication and release of raw data.[9] Other investigators may fear that future researchers will undermine the original authors' conclusions, either by uncovering an error or by employing alternative analytic methods. We believe that it is in the best interests of science to have a robust debate on the merits of particular research findings. However, it is only right and fair that investigators should have a say in the use of their data, so we have previously recommended[9] that original investigators should be included as co-authors on any publication resulting from re-analysis of raw data or, alternatively, be offered the opportunity to provide a response and commentary.

Some of the authors we contacted claimed that it would take “too much work” to provide us with raw data. This suggests that researchers do not always develop a clean, well annotated data set for analyses associated with a particular scientific paper. This strikes us as a problem in and of itself. We also found that that, as time passes from the date of publication, authors sometimes lose access to the original data or switch institutions and without maintaining their the email address listed on the paper. A simple solution to both of these problems would be to require authors to submit de-identified data sets to journals or public repositories at the time of publication.

Data was requested from articles published in only two journals, PLoS Medicine and PLoS Clinical Trials, and it is possible that authors who publish in other journals are more likely to share data sets. This seems unlikely as open access journals with explicit data sharing policies are likely to attract authors who support greater openness to scientific data. Our method of requesting data sets was intentionally left vague as we were interested as much in the investigators responses as acquiring the actual data set; perhaps a more detailed request would have garnered more positive responses. Again this is unlikely, as no authors claimed that our request was unreasonable, inappropriate, or lacking in sufficient details, and further information was provided upon request. A final limitation was that our sample was small. Yet the results were so striking – only one in ten authors complied - that we can be fairly confident the true rate of compliance is far from 100%. Indeed, it would be highly unlikely to obtain only 1 in 10 data sets if the true rate of data sharing was even as low as 50% (p = 0.01 from binomial test).

In conclusion, our findings suggest that explicit journal policies requiring data sharing do not lead to authors making their data sets available to independent investigators.
